# Anti-5′-Nucleotidases (5′-ND) and Acetylcholinesterase (AChE) Activities of Medicinal Plants to Combat *Echis carinatus* Venom-Induced Toxicities

**DOI:** 10.1155/2021/6631042

**Published:** 2021-02-04

**Authors:** Nazia Aslam, Syeda Fatima, Sofia Khalid, Shahzad Hussain, Mughal Qayum, Khurram Afzal, Muhammad Hassham Hassan Bin Asad

**Affiliations:** ^1^Department of Environmental Sciences, Fatima Jinnah Women University, Rawalpindi, Pakistan; ^2^Drugs Control & Traditional Medicines Division, National Institute of Health, Islamabad, Pakistan; ^3^Department of Pharmacy, Kohat University of Science and Technology, Kohat 26000, Pakistan; ^4^Institute of Food Sciences and Nutrition, Bahauddin Zakariya University, Multan, Pakistan; ^5^Department of Pharmacy, COMSATS University Islamabad, Abbottabad Campus 22060, KPK, Pakistan; ^6^Institute of Fundamental Medicine and Biology, Department of Genetics, Kazan Federal University, Kazan 420008, Russia

## Abstract

*Echis carinatus* is one of the highly venomous snakes of Pakistan that is responsible for numerous cases of envenomation and deaths. In Pakistan, medicinal plants are commonly used traditionally for snakebite treatment because of their low cost and easy availability in comparison with antivenom. The current research is aimed at evaluating the inhibitory activity of Pakistani medicinal plants against acetylcholinesterase and 5′-nucleotidases present in *Echis carinatus* venom. Acetylcholinesterase and 5′-nucleotidase enzymatic assays were performed at different venom concentrations to check the activity of these enzymes. Methanolic extracts from different parts of plants were used for in vitro determination of their inhibitory activity against 5′-nucleotidases in snake venom. Active methanolic extracts were subsequently fractioned using different solvents, and these fractions were also assessed for their anti-5′-nucleotidase activity. Results of this study exhibited that *Eugenia jambolana* Willd. ex O. Berg, *Rubia cordifolia* L., *Trichodesma indicum* (L.) R. Br., *Calotropis procera* (Wild.) R. Br., *Curcuma longa* L., and *Fagonia arabica* L. were able to significantly (*p* > 0.5) neutralize the 5′-nucleotidase activity by 88%, 86%, 86%, 85%, 83.7%, and 83%, respectively, compared with a standard antidote (snake venom antiserum). Thus, this study indicates that these plants possess the potential to neutralize one of the toxic enzymatic components of *Echis carinatus* venom and hence can help to augment the future efforts of developing alternative therapy for the management of snakebites.

## 1. Introduction

Snake envenomation is a global medical problem that has always had serious implications for the health and welfare of human beings [1]. It is specifically more prevalent in the poor and rural regions of South and Southeast Asia, Latin America, sub-Saharan Africa, and Papua New Guinea [2, 3]. South Asia is predominantly the most affected region which experiences approximately 121,000 cases of envenoming and 14,000 deaths each year. This densely populated region is a hotspot of venomous snake species. Sociodemographic as well as occupational profile of people has major contribution in increasing the risk of human and snake interactions. In addition, inadequate first aid along with delayed and suboptimal treatment of snakebites has further aggravated the situation in this region [3–5].

*Echis carinatus* is one of the four main medically imperative snakes (referred to as “Big Four”) in South Asia [6], which is responsible for numerous cases of morbidity and mortality in the Indian subcontinent including Pakistan (Astola Island in Makran) [7]. Envenomation caused by *Echis carinatus* is mainly characterized by severe local tissue destruction which includes blisters, edema, myonecrosis, and hemorrhage, as well as systemic effects such as hemorrhage of the body's vital organs, hypertension, and disturbed hemostasis [8, 9]. Transcriptome investigation of the venom gland of genus *Echis* has shown the presence of various enzymes including L-amino oxidases, phospholipase A_2_, serine proteinases, snake venom metalloproteinase, 5′-nucleotidase, phosphomonoesterase, and phosphodiesterase [10, 11].

5′-Nucleotidases are ubiquitously present enzymes in snake venom. These are hydrolytic enzymes that have an important role in envenomation of prey/victim. 5′-Nucleotidases induce inhibition of platelet aggregation through their interaction with factor IX of the blood coagulation cascade. Factor IX is reported to be involved in normal blood clot formation; however, any disruption of this protein may lead to delay in blood coagulation. Its activation occurs on phospholipid membranes; however, activated factor VII (extrinsic pathway) and XI (intrinsic pathway) are required in the presence of Ca^++^ ions [12]. They further act on adenosine monophosphate and release purines, mainly adenosine, which assist in immobilization of prey [12]. Hypotension and paralysis induced by snake venom are believed to be brought about by purines through purine receptors [13]. Adenosine can also help to diffuse venom toxins in tissues by means of vasodilation and/or inhibition of platelet aggregation. Consequently, adenosine also acts as a potential spreading agent [14–16]. Additionally, adenosine is also known to inhibit the release of neurotransmitters in central and peripheral nerves, which results in paralysis [13, 17, 18]. Hence, adenosine has a central role in the envenomation strategies involved in immobilization of prey [13]. Snake venom is a rich source of acetylcholinesterase enzyme where it exists as a soluble, hydrophilic monomer. Venom acetylcholinesterase is more vigorous in terms of hydrolyzing acetylcholine than that found in Torpedo fish and mammals [19]. It has been hypothesized that the elevated level of acetylcholinesterase enzyme in snake venom principally affects the nervous system through disruption of cholinergic transmission both at the neuromuscular junction and in the central nervous system [20].

Currently, animal-derived snake venom antiserum is the only effective therapy for treatment of snakebites [21]. Drawbacks of conventional antivenom include adverse allergic reactions, inability to efficiently neutralize all clinical symptoms, and complex production processes that involve animal husbandry and extraction of snake venom. Timely transportation to rural areas is another problem as they usually lack proper medical facilities to stock and administer antivenom [22, 23]. In this scenario, medicinal plants could be a viable and less costly option. Pakistan has a range of climatic and geographical zones with rich plant biodiversity. There are approximately 6000 species of plants [24, 25], of which about 400-600 are used for medicinal purposes [26]. Evaluating medicinal plants for antisnake venom activity could be beneficial as they have long been used traditionally for the treatment of snakebites, specifically in remote areas without a proper healthcare system [27]. The current study was therefore carried out to evaluate the neutralizing potential of Pakistani medicinal plants against 5′-nucleotidases and acetylcholinesterase enzymes present in *Echis carinatus* venom.

## 2. Materials and Methods

### 2.1. Snake Venom and Chemicals

Lyophilized venom of *Echis carinatus* was kindly given by the National Institutes of Health (NIH), Islamabad, Pakistan. Venom was stored at 2 to 8°C in a light-resistant bottle. All other chemicals were purchased from Merck unless otherwise described.

### 2.2. Medicinal Plants

Plants having ethnobotanical evidence for antisnake venom activity were selected for this study. These plants were collected from various regions of Pakistan, while few were acquired from a local market in Rawalpindi. Plants were identified by an expert plant taxonomist, and voucher specimens were submitted to the herbarium of the Department of Botany, Bahauddin Zakariya University, Multan, Pakistan. Details of selected medicinal plants have been presented in Table 1.

### 2.3. Plant Extraction Process

After thoroughly washing, shade drying, and chopping, different parts of the pants were soaked in methanol for a period of about four weeks at ambient temperature. After that, the filtration process was carried out firstly with ordinary filter paper and then using Whatman filter paper 41. Subsequently, plant extracts were dried and stored in amber glass vials at 8°C in a refrigerator [33].

### 2.4. Enzymatic Assay for Acetylcholinesterase

Acetylcholinesterase activity in *Echis carinatus* venom was assessed using acetylthiocholine iodide as a substrate. Briefly, the reaction mixture containing venom (1–8 mg), phosphate buffer (pH 8.0), 10 mmol DTNB (5,5′-dithiobis(2-nitrobenzoic acid)), and acetylthiocholine iodide was incubated at 37°C for a period of 10 min. Hydrolysis of acetylthiocholine iodide by acetylcholinesterase enzyme of snake venom is depicted by the appearance of yellow color, which is produced due to the reaction of thiocholine with DTNB. The amount of yellow color produced was measured at 412 nm using a spectrophotometer (UV-1280 by Shimadzu) [36–38].

### 2.5. Enzymatic Assay for 5′-Nucleotidases

To perform the 5′-nucleotidase assay, adenosine 5′-monophosphate (5′-AMP) was used as substrate. Concisely, reaction mixture containing 5′-AMP (0.02 M, 0.5 mL), glycine buffer (0.2 M, 0.5 mL), magnesium sulfate (0.1 M, 0.1 mL), and venom (10-40 *μ*g) was incubated for 10 minutes at 37°C. After that, the reaction was stopped by adding 1.5 mL of 10% trichloroacetic acid (TCA). The concentration of inorganic phosphate released in the reaction mixture was analyzed using the ascorbic acid reagent by adopting the protocol as described by Tan et al. [39]. The reaction mixture was allowed to stand at room temperature for 30 min, and absorbance was then measured at 820 nm. A standard curve was also constructed using known concentrations of inorganic phosphate [39]. For evaluation of anti-5′-nucleotidase activity, venom was preincubated with plant extracts at 37°C for 15 min [40].

### 2.6. Fractionation of Active Plant Extracts

Fractionation of active methanolic plant extracts (they have shown antienzymatic activity in their crude form of extract) was carried out using four different solvents, *i.e.*, n-hexane, chloroform, dichloromethane, and ethyl acetate, based on their ascending polarity, respectively (relevant constituents dissolved in their relevant polarity of solvents) [41, 42]. These fractions, after filtration and drying, were again tested for their inhibitory activity against 5′-nucleotidase enzymes of *Echis carinatus* venom.

### 2.7. Phytochemical Analysis

Active methanolic plant extracts as well as their active fractions were analyzed qualitatively for the presence of different phytochemical constituents using standard procedures [43].

### 2.8. Statistical Analysis

All results were expressed as the mean. The Student *t*-test (SPSS) was used to compare the significance of the experimental results with the standard antidote (snake venom antiserum). The level of significance was set at *p* > 0.5.

## 3. Results

Acetylcholinesterase enzyme induces hydrolysis of acetylcholine which results in the liberation of choline and acetic acid. The acetylcholinesterase assay was performed using acetylthiocholine iodide as a substrate. Different concentrations of *Echis carinatus* venom were used to check the activity of the acetylcholinesterase enzyme. Results indicate that there was no significant increase in acetylcholinesterase activity with the increase in venom concentration. Even at 8 mg venom dose, very low acetylcholinesterase activity (0.0808 units/mg) was observed in snake venom (Table 2). Hence, it can be said that *Echis carinatus* venom contains a very low amount of acetylcholinesterase enzyme. So, this assay was rejected and not proceeded further.

**Figure 1:**
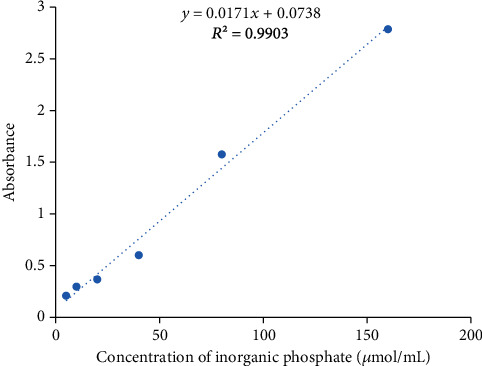
Standard curve for inorganic phosphate to generate a standard curve.

5′-Nucleotidases induced hydrolytic cleavage of adenosine monophosphate, which results in the liberation of inorganic phosphate. A standard curve was constructed with a known concentration of inorganic phosphate (Figure 1). 5′-Nucleotidase activity was assessed at different concentrations of *Echis carinatus* venom. Enzymatic activities at venom concentrations of 10 *μ*g, 20 *μ*g, 30 *μ*g, and 40 *μ*g were found to be 145, 223, 303, and 386 units/mg, respectively (Table 3). A fixed venom concentration (10 *μ*g) was then used to evaluate the inhibitory potential of Pakistani medicinal plants against 5′-nucleotidase enzymes of *Echis carinatus* venom. In this study, snake venom antiserum was used as the reference standard.

Results showed that among eighteen selected medicinal plants, six plants were able to significantly neutralize the 5′-nucleotidase activity of *Echis carinatus* venom. Maximum inhibition was shown by *Eugenia jambolana* Willd. ex O. Berg (88%, *p* > 0.5), followed by *Rubia cordifolia* L. (86%, *p* > 0.5), *Trichodesma indicum* (L.) R.Br. (86%, *p* > 0.5), *Calotropis procera* (Wild.) R.Br. (85%, *p* > 0.5), *Curcuma longa* L. (83.7%, *p* > 0.5), and *Fagonia arabica* L. (83%, *p* > 0.5). Other plants show moderate to low anti-5′-nucleotidase activities. Inhibitory activities of all medicinal plants against 5′-nucleotidase enzymes have been given in Table 4. Fractions of active plant extracts were also analyzed for their neutralizing potential against 5′-nucleotidase enzymes of *Echis carinatus* venom. Inhibitory activity of different fractions of active methanolic plant extracts has been shown in Table 5.

Fractionation results showed that all four fractions of *Eugenia jambolana* Willd. ex O. Berg showed inhibitory activities comparable to the crude extract which were as follows: n-hexane 86.8%, chloroform 88%, dichloromethane 80.5%, and ethyl acetate 90%. In the case of *Calotropis procera* (Wild.) R.Br., two fractions were effective; chloroform fraction inhibited 5′-nucleotidase activity by 84% and dichloromethane fraction by 76.5%. For *Curcuma longa* L., dichloromethane fraction (85%), and for *Fagonia arabica* L., ethyl acetate fraction (85.5%), showed inhibition close to the crude extract. Dichloromethane and ethyl acetate fractions of *Rubia cordifolia* L. exhibited 89% and 88% inhibition, respectively. For *Trichodesma indicum* (L.) R. Br., only chloroform fraction showed percentage inhibition (88%) comparable to the crude extract. Phytochemical screening was also performed for active methanolic plant extract as well as their active fractions. Phytochemical analysis results have been presented in Tables 6–10.

## 4. Discussion

In Pakistan, the increased frequency of snakebites is usually attributed to the destruction of snakes' habitats and subsequent migration of these venomous animals to human settlements [44]. *Echis carinatus* is one of the highly venomous snakes in South Asia including Pakistan that is responsible for more bites and deaths among the human population than any other snake species [45]. 5′-Nucleotidases are one of the enzymatic components of *Echis carinatus* venom [6, 11]. 5′-Nucleotidase enzymes act as a cofactor of hemorrhagic toxins and affect homeostasis through modulation of platelet function [13, 46, 47]. They have been described as the most potent platelet aggregation inhibitors [48]. Inhibition of platelet aggregation caused by 5′-nucleotidase enzymes subsequently leads to inhibition of blood coagulation [49]. Snake venom acetylcholinesterase is quite stable compared to acetylcholinesterase enzymes from other sources. It is believed that this enzyme principally affects the nervous system of prey/victim through disruption of cholinergic transmission [20].

Medicinal plants used for the treatment of snakebites are commonly found worldwide, particularly in the regions of Asia, Africa, and America [50–52]. In developing countries like Pakistan, the advanced allopathic medication framework is either exorbitant or lacking, so people in rural areas are mostly dependent on plants for primary healthcare [53]. Many indigenous communities use plants as an alternative remedy in an attempt to treat or reduce the toxic effects of snake venom like edema and hemorrhage [54]. Plant extracts together with their fractions and isolates have been reported to have neutralizing ability against snake venom as well as its purified toxins. These plant-based inhibitors not only decrease the local tissue destruction but also help to delay the systemic diffusion of venom toxins and, hence, assist in increasing the survival time of snakebite victims [51, 55]. Several mechanisms have been proposed for the inactivation of snakes' venom by plants. Nevertheless, two main mechanisms that have been anticipated to be involved in neutralization of venom components by plants include enzyme inhibition and precipitation of protein [54]. Snake venom enzymes are the key components involved in venom toxicity. Hence, inactivation of venom enzymes is generally thought to be the fundamental step in snakebite management [56]. So, the present study is an attempt to assess the activity of acetylcholinesterase and 5′-nucleotidase enzymes in *Echis carinatus* venom as well as their neutralization by Pakistani medicinal plants.

In this study, *Echis carinatus* venom showed very low activity of acetylcholinesterase enzyme. Similar results were reported by a previous study where significant activity of acetylcholinesterase enzyme was found in the elapid venom while no acetylcholinesterase activity was detected in the viperid venom [57]. Another study conducted by Hashmi et al. [6] also revealed very low or no activity of acetylcholinesterase enzyme in the venom of *Echis carinatus* and *Daboia russelii*, whereas considerable activity was observed in the venom of *Bungarus caeruleus* and *Naja naja.* Observations of aforementioned studies reveal that snakes belonging to the family Viperidae contain a low or negligible amount of acetylcholinesterase enzyme in their venom. Accordingly, *Echis carinatus*, being the member of the family Viperidae, showed extremely low acetylcholinesterase activity.

5′-Nucleotidase activity was observed in a dose-dependent manner in Pakistani *Echis carinatus* venom. Inhibition study results revealed that, among eighteen medicinal plants, six plants showed anti-5′-nucleotidase activity comparable to standard antidote (*p* > 0.5). Some fractions of these active plant extracts also showed noteworthy inhibitory activity against 5′-nucleotidases present in snake venom. Previous studies have also reported such neutralizing ability of medicinal plants against nucleotidase activity of *Echis carinatus* venom. A study showed that different extracts of the *Tabernaemontana alternifolia* root (ethyl acetate, acetone, ethanol, methanol, and water) were able to completely inhibit the 5′-nucleotidase activity of *Echis carinatus* venom [58]. In another in vitro study, methanolic extract of the *Canthium parviflorum* root showed promising inhibitory activity against 5′-nucleotidase enzymes of *Echis carinatus* venom [59]. Plants' secondary metabolites possess the ability to neutralize a range of enzymes present in snake venom such as 5′-nucleotidases, protease, phospholipase A_2_, L-amino acid oxidase, and hyaluronidase. Various bioactive compounds have been reported in literature for their ability to inhibit one or more snake venom enzymes, for example, resveratrol [60, 61], *β*-sitosterol [62–64], pentagalloyl glucopyranose [31, 65], gallic acid [66, 67], solanidane [68], alternamin [69], macrolobins A and B [70], and 8-methoxy coumestrol [71]. In this study, phytochemical screening of active fractions of plants' extracts showed the presence of various bioactive compounds such as flavonoids, phenols, saponins, tannins, terpenoids, steroids, and alkaloids. Hence, anti-5′-nucleotidase activities may be attributed to these different phytochemicals present in selected medicinal plants. In this regard, future studies regarding the identification of specific components of these plant extracts can be valuable to augment the efforts of developing an alternative therapy to combat the toxic effects of snake envenomation.

## 5. Conclusion

This study revealed that *Calotropis procera* (Wild.) R. Br., *Curcuma longa* L., *Eugenia jambolana* Willd. ex O. Berg, *Fagonia arabica* L., *Rubia cordifolia* L., and *Trichodesma indicum* (L.) R.Br. possess the ability to neutralize the 5′-nucleotidase enzymes present in Pakistani *Echis carinatus* venom. So, based on this study, it can be concluded that these plants can serve as the potent source of bioactive compounds with antivenom property for managing the toxicities of snakebites, particularly the effects of 5′-nucleotidase enzymes which are the potent inhibitor of platelet aggregation in victims.

## Figures and Tables

**Table 1:** List of medicinal plants used to evaluate inhibitory activity against 5′-nucleotidases of *Echis carinatus* venom.

Sr. no.	Medicinal plants (voucher number)	Family	Part used	References
1	*Adiantum capillus-veneris* L. (R.R.Stewart F.W.Pak.4(2))	Pteridaceae	Whole plant	[28]
2	*Albizia lebbeck* (L.) Benth. (R.R.Stewart F.W.Pak.381(9))	Fabaceae	Seeds	[28]
3	*Althaea officinalis* L. (R.R.Stewart F.W.Pak.477(6))	Malvaceae	Roots	[29]
4	*Calotropis procera* (Wild.) R.Br. (R.R.Stewart F.W.Pak.566(6))	Apocynaceae	Flower	[28]
5	*Citrullus colocynthis* (L.) Schrad. (R.R.Stewart F.W.Pak.702(10))	Cucurbitaceae	Fruit	[28]
6	*Curcuma longa* L. (R.R.Stewart F.W.Pak.66(3))	Zingiberaceae	Rhizome	[30]
7	*Eclipta prostrata* (L.) L.Mint (R.R.Stewart F.W.Pak.743(5))	Asteraceae	Whole plant	[28]
8	*Eugenia jambolana* Willd. ex O. Berg (R.R.Stewart F.W.Pak.504(2))	Myrtaceae	Fruit	[28]
9	*Fagonia arabica* L. (R.R. Stewart F.W. Pak.433(2))	Zygophyllaceae	Leaves and twigs	[31]
10	*Lepidium sativum* L. (R.R. Stewart F.W.Pak.319(4))	Brassicaceae	Whole plant	[32]
11	*Matthiola incana* (L.) R.Br. (R.R. Stewart F.W.Pak.322(2))	Brassicaceae	Seeds	[33]
12	*Momordica charantia* L. (R.R. Stewart F.W. Pak.706(1))	Cucurbitaceae	Fruits	[30]
13	*Psoralea corylifolia* L. (R.R. Stewart F.W. Pak.418(1))	Fabaceae	Seeds	[33]
14	*Rubia cordifolia* L. (R.R. Stewart F.W. Pak.689(4))	Rubiaceae	Roots	[28]
15	*Sapindus mukorossi* Gaertn. (R.R. Stewart F.W. Pak.463(3))	Sapindaceae	Fruits	[28]
16	*Swertia chirayita* (Roxb.ex Flem.) Karst. (R.R.Stewart F.W.Pak.561(4))	Gentianaceae	Stems	[34]
17	*Terminalia arjuna* Wight & Arn (R.R. Stewart F.W.Pak.502(4))	Combretaceae	Bark	[28]
18	*Trichodesma indicum* (L.) R.Br. (R.R. Stewart F.W.Pak.604(3))	Boraginaceae	Leaves	[35]

**Table 2:** Enzymatic activity of acetylcholinesterase at different concentrations of *Echis carinatus* venom.

Concentration of venom used	Absorbance (*mean* ± *S*.*D*.)	Enzyme activity (units/mg)
8 mg	0.045 ± 0.0092	0.0808
4 mg	0.044 ± 0.0102	0.0790
2 mg	0.039 ± 0.0141	0.0701
1 mg	0.030 ± 0.0078	0.0539

**Table 3:** Enzymatic activity of 5′-nucleotidases at different concentrations of Echis carinatus venom.

Concentration of venom used	Absorbance (*mean* ± *S*.*D*.)	Enzyme activity (units/mg)
0.04 mg	2.712 ± 0.2202	386
0.03 mg	2.149 ± 0.1307	303
0.02 mg	1.601 ± 0.1594	223
0.01 mg	1.063 ± 0.1296	145

**Table 4:** Inhibitory activity of medicinal plants (10 *μ*g/0.1 mL) evaluated against 5′-nucleotidase enzymes (10 *μ*g/0.1 mL) of *Echis carinatus* venom.

Sr. no.	Evaluated samples		5′-Nucleotidase activity (units/mg)	Inhibition (%)	Statistics
1	*Adiantum capillus-veneris* L.		127	12^∗∗^	*p* > 0.001
2	*Albizia lebbeck* (L.) Benth.		119	17.8^∗∗^	*p* > 0.001
3	*Althaea officinalis* L.		94	35^∗∗^	0.05 > *p* > 0.01
4	*Calotropis procera* (Wild.) R.Br.		21.5	85^∗^	*p* > 0.5
5	*Citrullus colocynthis* (L.) Schrad.		100.7	30.6^∗∗^	*p* > 0.001
6	*Curcuma longa* L.		23.6	83.7^∗^	*p* > 0.5
7	*Eclipta prostrata* (L.) L.Mint		43	70^∗∗^	*p* > 0.001
8	*Eugenia jambolana* Willd. ex O. Berg		17	88^∗^	*p* > 0.5
9	*Fagonia arabica* L.		24.5	83^∗^	*p* > 0.5
10	*Lepidium sativum* L.		114	21^∗∗^	*p* > 0.001
11	*Matthiola incana* (L.) R.Br.		116.5	19.6^∗∗^	*p* > 0.001
12	*Momordica charantia* L.		76	47^∗∗^	0.05 > *p* > 0.01
13	*Psoralea corylifolia* L.		114	21^∗∗^	*p* > 0.001
14	*Rubia cordifolia* L.		20	86^∗^	*p* > 0.5
15	*Sapindus mukorossi* Gaertn.		56	61^∗∗^	0.05 > *p* > 0.01
16	*Swertia chirayita* (Roxb.ex Flem.) Karst.		37	74^∗∗^	0.5 > *p* > 0.1
17	*Terminalia arjuna* Wight & Arn		57	60.5^∗∗^	0.1 > *p* > 0.05
18	*Trichodesma indicum* (L.) R.Br.		20	86^∗^	*p* > 0.5
19	Standard inhibitor of 5′-nucleotidase enzyme	Snake venom antiserum	17.7	87.8^∗∗∗^	Used for comparison of experimental results

Note: ∗ indicates *p* values nonsignificantly different from the standard antidote. ∗∗ indicates *p* values significantly different from the standard antidote. ∗∗∗ indicates value selected to compare the results.

**Table 5:** Anti-5′-nucleotidase activity of different fractions (10 *μ*g/0.1 mL) of active methanolic plant extracts.

Sr. no.	Active plant extracts	Fractions (10 *μ*g/0.1 mL)	5′-Nucleotidase activity (units/mg)	Inhibition (%)
1	*Calotropis procera* (Wild.) R.Br.	n-Hexane	74	48.7
		Chloroform	23	84
		Dichloromethane	34	76.5
		Ethyl acetate	67.7	53

2	*Curcuma longa* L.	n-Hexane	122	15.7
		Chloroform	82	43
		Dichloromethane	21.5	85
		Ethyl acetate	57	60.6

3	*Eugenia jambolana* Willd. ex O. Berg	n-Hexane	19	86.8
		Chloroform	17	88
		Dichloromethane	28	80.5
		Ethyl acetate	14	90

4	*Fagonia arabica* L.	n-Hexane	99.6	31
		Chloroform	128	11
		Dichloromethane	68.9	52.5
		Ethyl acetate	21	85.5

5	*Rubia cordifolia* L.	n-Hexane	105.8	27
		Chloroform	109	24.7
		Dichloromethane	15.6	89
		Ethyl acetate	17	88

6	*Trichodesma indicum* (L.) R. Br.	n-Hexane	126.6	12.7
		Chloroform	17	88
		Dichloromethane	56	61
		Ethyl acetate	85	41

**Table 6:** Qualitative analysis of phytochemicals in the crude extracts of *Calotropis procera* (Wild.) R. Br., *Curcuma longa* L., and *Eugenia jambolana* Willd. ex O. Berg.

Phytochemicals	*Calotropis procera* (Wild.) R. Br.	*Curcuma longa* L.	*Eugenia jambolana* Willd. ex O. Berg
Alkaloids	+	+	+
Carbohydrates	+	−	−
Fatty acids	−	−	−
Flavonoids	+	+	+
Glycosides	+	−	+
Phenols/tannins	+	+	+
Proteins	+	−	−
Saponins	+	+	+
Terpenoids/steroids	+	+	+

Note: (+) indicates the presence and (−) indicates the absence of phytochemicals.

**Table 7:** Qualitative analysis of phytochemicals in the crude extracts of *Fagonia arabica* L., *Rubia cordifolia* L., and *Trichodesma indicum* (L.) R. Br.

Phytochemicals	*Fagonia arabica* L.	*Rubia cordifolia* L.	*Trichodesma indicum* (L.) R. Br.
Alkaloids	+	+	+
Carbohydrates	−	−	−
Fatty acids	−	−	−
Flavonoids	+	+	+
Glycosides	+	+	−
Phenols/tannins	+	+	+
Proteins	−	+	−
Saponins	+	+	+
Terpenoids/steroids	+	+	+

Note: (+) indicates the presence and (−) indicates the absence of phytochemicals.

**Table 8:** Qualitative analysis of phytochemicals in the active fractions of *Calotropis procera* (Wild) R. Br. and *Curcuma longa* L. crude extracts.

Phytochemicals	*Calotropis procera* (Wild) R. Br.		*Curcuma longa* L.
	Chloroform	Dichloromethane	Dichloromethane
Alkaloids	+	+	+
Carbohydrates	−	−	−
Fatty acids	−	−	−
Flavonoids	+	+	+
Glycosides	−	−	−
Phenols/tannins	+	+	+
Proteins	−	−	−
Saponins	+	−	+
Terpenoids/steroids	−	+	+

Note: (+) indicates the presence and (−) indicates the absence of phytochemicals.

**Table 9:** Qualitative analysis of phytochemicals in active fractions of *Eugenia jambolana* Willd. ex O. Berg crude extract.

Phytochemicals	*Eugenia jambolana* Willd. ex O. Berg			
	n-Hexane	Chloroform	Dichloromethane	Ethyl acetate
Alkaloids	+	+	+	+
Carbohydrates	−	−	−	−
Fatty acids	−	−	−	−
Flavonoids	+	+	+	+
Glycosides	+	+	+	+
Phenols/tannins	−	+	−	+
Proteins	−	−	−	−
Saponins	+	+	+	+
Terpenoids/steroids	−	+	−	+

Note: (+) indicates the presence and (–) indicates the absence of phytochemicals.

**Table 10:** Qualitative analysis of phytochemicals in the active fractions of *Fagonia arabica* L., *Rubia cordifolia* L., and Trichodesma indicum (L.) R. Br. crude extracts.

Phytochemicals	*Fagonia arabica* L.	*Rubia cordifolia L.*		*Trichodesma indicum* (L.) R. Br.
	Ethyl acetate	Dichloromethane	Ethyl acetate	Chloroform
Alkaloids	+	+	+	+
Carbohydrates	−	−	−	−
Fatty acids	−	−	−	−
Flavonoids	+	+	−	+
Glycosides	−	−	−	+
Phenols/tannins	+	+	+	+
Proteins	−	+	−	+
Saponins	+	+	−	−
Terpenoids/steroids	+	+	+	+

Note: (+) indicates the presence and (−) indicates the absence of phytochemicals.

## Data Availability

Data used to support this study finding have been included in the article and could be provided upon request from first author Nazia Aslam (nazia.3284@gmail.com).
